# Before the Lords' Committee

**Published:** 1890-07-26

**Authors:** 


### BEFORE THE LORDS' COMMITTEE.

(BY OUR SPECIAL COMMISSIONER.)

### VIII.—THE NURSES OF THE LONDON HOSPITAL.

ON the 14th inst. Mr. Carr-Gomm, late of the Madras Civil Service, and chairman of the London Hospital from 1885 to 1889, gave evidence. He said the matron had no power to dismiss nurses or sisters, but she could, according to a new arrangement, terminate a probationer's agreement during the first year, if she considered the probationer was unsuited for nursing work. The Committee had frequently enquired into cases of dismissal with the result that they always confirmed the matron's decision. The full report of nurse Page's dismissal and the subsequent enquiry was read. Asked if, in spite of the recent allegations, he was quite satisfied with the present system of nursing at the London Hospital, Mr. Carr-Gomm answered emphatically in the affirmative, and stated that the Committee had perfect confidence in their matron. They had put the case of the probationers into her hands and had never repented the course. A letter from Mrs. Macdonald was read in which she stated that as Sister of the George Wards she considered probationers Yatman and Raymond had "mistaken their vocation" as she found them unsatisfactory as nurses. The salaries of certain of the officials were stated to be—house governor, £900; secretary, £400; matron, £250; chaplain, £250. Probationers always could complain to the committee or the visitors for the week if they were in any way dissatisfied; as a matter of fact, the matron never let a probationer leave the hospital till after the meeting of the House Committee, so that all had opportunity to appeal if they desired it. Asked his opinion about a resident medical officer, Mr. Carr-Gomm said he preferred the present plan. With regard to the late chaplain (Mr. Valentine), Mr. Carr-Gomm stated: "He told me on leaving he should not leave a stone unturned to do the hospital and the committee an injury; therefore, I cannot help thinking his evidence was biassed." This announcement caused some sensation in the crowded committee room, as did also the full account of Mr. Valentine's views on confession. The Archbishop of Canterbury was present as one of the Committee, and evidently followed this evidence with interest.

Miss Manley, late night sister of the London Hospital, was then called in reference to a sensational story told by Probationer Homersham. Miss Manley stated: "I never saw Dr. —— the worse for drink; I never refused him a hypodermic syringe; I never encouraged a nurse to hide a syringe." By Lord Sandhurst: "You contradict the statement of Miss Homersham?"—"I contradict it emphatically; it is absolutely without foundation." Miss Manley, who gave her evidence very clearly and quietly, further said she did not consider the nurses' work at the London excessive, nor had she heard much grumbling.

Mr. Weatherall, late house physician to Sir Andrew Clark, contradicted the statement that the beds were wheeled out of one ward into another, in order to deceive the visiting physicians as to the crowded state of the wards.

Mr. Brookes, late assistant chaplain, brought forward the case of Nurse Howes, said to have been unfairly dismissed. Miss Lückes went into the box and explained the circumstances to the Committee.

On the 17th inst. Mr. Valentine desired to make a personal statement. It was untrue that he said, on leaving the London Hospital, that he should try to injure that institution.

The Archbishop of Canterbury called attention to a minor point with regard to answer 6,929, which he said implied that Mr. Valentine submitted his letter to the London House Committee to him before sending it to the Committee—"it was not submitted to me till after," concluded the Archbishop.

Dr. Buksh was then called and stated he was house physician and surgeon at the London Hospital in '84 and '85. He emphatically denied statements 5,809, 5,882, 5,889, and 5,892, made by Miss Homersham; they were absolutely false. He denied that he was ever drunk on duty; he denied that he would want to administer morphia to a kidney case; and he never used insulting language to any nurse in the hospital.

Mrs. Perry, wife of the warden of Guy's, and for three-and-a-half years a sister at the London Hospital stated that the nurses at the London were justly treated; special nurses were always supplied when wanted; her patients never suffered from the frequent changing of the nurses; and she considered the staff sufficient.

Dr. Fenwick, who has been connected with the London Hospital for twenty-two years, stated that he and Dr. Sutton were responsible for the sick nurses; that the house physicians and surgeons were not "boys" but picked men, all holding double qualifications, and their average age was 27. Dr. Fenwick said he could not imagine what those probationers were talking about who said that their health was in the charge of "boys;" surely he and Dr. Sutton were old enough. (Laughter). Dr. Fenwick gave it as his opinion that it would be very much better to have a resident medical officer in every hospital, that officer to be under the control of the visiting staff when they were there, and over the house physicians and surgeons in the absence of the visiting staff. Dr. Fenwick also stated that he considered the present system of medical education all wrong; he was in favour of one general university, and then certain hospitals registered for instruction. He should like all lecturers and visiting physicians and surgeons to be paid; this was usual abroad. Every hospital and every workhouse infirmary could and ought to be used for clinical teaching; there ought to be a visiting staff to every workhouse infirmary. The wards of the London Hospital were crowded; the district from which it drew its patients was enormous, and it was a very poor hospital. But it was not true that beds were wheeled from one ward to another at the time that Sir Andrew Clark was expected, as stated by probationer Homersham. "That witness must have been dreaming. I was Sir Andrew Clark's assistant, and it was never done," said Dr. Fenwick. With regard to the nurses of the London Hospital, they were capital nurses, and he had never heard a complaint against them. He always told outside physicians "If you want good nurses, go to the London." He should like the nurses to have three weeks' holiday in the year, and shorter hours. He did not consider, however, that they were overworked, and he had never known his patients neglected. The nurses were sufficiently well fed. He had never had to complain of the male nurses.

Mr. Frederick Treves, F.R.C.S., next gave emphatic evidence in favour of the nurses and nursing of the London Hospital. Mr. Treves has been connected with the hospital for 19 years, is lecturer to the nurses, and has care of their surgical ailments. He said, "So far as the surgical cases are concerned the charges brought are exceedingly unjust. No nurse has ever passed through a surgical ailment without being seen by me. The statement that the visiting staff does not visit the sick-room is also unfounded." Mr. Treves said the work of the nurses was intensely hard, but many women took up nursing who were physically unfit for any sort of work. The bulk of the nurses were in perfect health, and he heard no complaints. He had had 93 nurses from the London Hospital for his private patients, 76 had had their full training, 17 were probationers. He had only had to complain of one. They were exceedingly well trained, and in his opinion unequalled by any other body of nurses. He had never noticed any inconvenience in his wards through nurses being sent out to private cases. Mr. Treves said he considered a central register for nurses would be detrimental to the nurses, the public, and the medical profession. Anything which tended to take the control from the training schools would be injurious, and a central register could not have that personal acquaintance with nurses the matrons of the training schools had. Registration would do away with all individuality; it would be like putting your hand in a basket, and pulling out the first apple which came, whether good or bad. It would be most injurious to the general practitioner if these sort of nurses held any diploma. Finally, Mr. Treves made a statement about the matron of the London Hospital; she was an exceptionally kind and considerate matron, which fact was testified by her wide popularity. Her extreme thoughtfulness had been conspicuous. Mr. Treves said he was in constant communication with the nurses, and could not understand how any could consider themselves aggrieved and not consult him. Mr. Valentine's statements about the sisters' rooms Mr. Treves stigmatised as "simply ludicrous, and quite impossible."

On the 21st inst. Miss Mackay gave it as her opinion that there was not a sufficient staff of nurses on night duty at the London Hospital; that the food of the nurses was unsatisfactory; and that the nurses were overworked. These points have been thrashed out before.

Miss Yatman contradicted the letter put in by Mr. Carr-Gomm.

Miss Lückes brought forward some figures as to the number of nurses and how they were employed; also as to the linen used in the wards. The number of paying probationers never exceeded thirteen. In very exceptional cases nurses were taken from the wards to be sent to private cases, but never if the nurse was wanted in the wards. Miss Lückes said she thought nurses should be much better paid, and should have, at least, four weeks' holiday in the year. They ought to have 2s. 6d. allowed them weekly for washing. The private institution in connection with the London was started because, in Miss Lücke's experience, the sick rich were not so well nursed as the sick poor; it was not started at first with the notion of making a profit for the hospital, though, as a fact, it had proved a profit. Convalescent homes were not necessary for nurses; when on a holiday they wanted change of surroundings. Miss Lückes found no difficulty in getting ladies to take in nurses who wanted change; if a nurse preferred it she was sent to a convalescent home at Brighton; even then the mingling with patients was better for her than mingling with fellow nurses and talking over cases. The present harmony between doctors and nurses resulted from each working on their own lines. Registration would probably introduce conflict between the nurse and the general practitioner. The ideal ward would contain only 30 beds, all under one physician, and nursed by a sister, two nurses, and two probationers on day duty, and one nurse and two probationers on night duty.

Mr. Nixon, house governor of the London Hospital, stated that he was responsible for all departments of the hospital save the secretarial, the matron's, and the chaplain's. His petty cash account came to about £10,000 per annum; it was all accounted for by vouchers. Mr. Nixon stated his methods of calculating the costs of out and in patients. Some of the Committee put questions to try and discover what became of the interest on the capital of the hospital, which they could not find accounted for in the published balance-sheet. Mr. Nixon agreed it would be better if all hospital accounts were kept on a common basis; he did not think a glossary was necessary for such a plan. He thought the hospitals would readily agree to keeping their accounts according to a common plan. The income of the London was £16,000, the expenditure £50,000; legacies were usually spent. In spite of the enormous difference between income and expenditure, there was seldom difficulty in raising funds; they did not tout. All books were put before the House Committee every week, and they frequently examined them. The contracts are for six months, and are made by the House Committee; the store-keeper is responsible for the bread, milk, and potatoes, the housekeeper for the meat, eggs, and groceries. There is a surveyor connected with the hospital who receives £200 per annum; he advises on the necessary repairs and superintends the carrying out of the same.

